# Genome-Wide DNA Methylome and Transcriptome Analysis of Porcine Testicular Cells Infected With Transmissible Gastroenteritis Virus

**DOI:** 10.3389/fvets.2021.779323

**Published:** 2022-01-13

**Authors:** Jiayun Wu, Xiaoru Shi, Lisi Wu, Zhengchang Wu, Shenglong Wu, Wenbin Bao

**Affiliations:** ^1^Key Laboratory for Animal Genetic, Breeding, Reproduction and Molecular Design of Jiangsu Province, College of Animal Science and Technology, Yangzhou University, Yangzhou, China; ^2^Joint International Research Laboratory of Agriculture and Agri-Product Safety, Yangzhou University, Yangzhou, China

**Keywords:** pig, TGEV, DNA methylation, gene expression, ST cells

## Abstract

Transmissible gastroenteritis virus (TGEV) is a porcine pathogen causing highly communicable gastrointestinal infection that are lethal for suckling piglets. In an attempt to delineate the pathogenic mechanism of TGEV-infected porcine testicular cells (ST cells), we conducted a whole genome analysis of DNA methylation and expression in ST cells through reduced bisulfate-seq and RNA-seq. We examined alterations in the methylation patterns and recognized 1764 distinct methylation sites. 385 differentially expressed genes (DEGs) were enriched in the viral defense and ribosome biogenesis pathways. Integrative analysis identified two crucial genes (*EMILIN2, RIPOR3*), these two genes expression were negatively correlated to promoter methylation. In conclusion, alterations in DNA methylation and differential expression of genes reveal that their potential functional interactions in TGEV infection. Our data highlights the epigenetic and transcriptomic landscapes in TGEV-infected ST cells and provides a reliable dataset for screening TGEV resistance genes and genetic markers.

## Introduction

DNA methylation is a widely studied mode of epigenetic modification, play a crucial role in modulating gene expression and chromatin conformation. Typically, DNA is methylated when methyl groups are added to the 5 'C cytosine position, in response to DNA methyltransferases (1). DNA methylation is regarded as a reliable and accessible epigenetic marker (2). Abnormally methylated DNA can induce diseases like dysplasia and tumors. Interestingly, pathogenic bacteria, drug therapy, and food supply can alter genomic methylation status, thereby manipulating the expression of responsive genes and facilitating phenotypic consequences (3–5). Therefore, the study of DNA methylation is crucial for the in-depth comprehension of gene expression, ontogeny, and disease development.

Transmissible gastroenteritis (TGE) is a highly communicable gastrointestinal disease caused by the transmissible gastroenteritis virus (TGEV), clinical symptoms such as vomiting, watery diarrhea, and severe dehydration. TGE was first reported in 1946 in the United States (6). It is particularly deadly due to its rapid onset, progression, and death within 1 to 2 days. It is also more prevalent in winter and spring, is highly contagious, and spreads rapidly especially under poor feeding conditions (7). Antibiotic treatment of this disease is ineffective. Hence, disease prevention and herd treatment are crucial for the enhancement of immunity and symptomatic treatment, respectively. TGEV presents a major challenge to the pig industry and has received recognition as a member of the 117 virulent infectious diseases by the World Organization for Animal Health (OIE) in 2018. TGEV is an enveloped virion with pleomorphism (mostly oval), which comes from the genus *Coronavirus* and family *Coronaviridae* (8). It targets the nutrient absorption capacity of porcine small intestine leading to reduced feed conversion rates (9). TGEV inoculation alters Na^+^ transport and accelerates extravascular protein loss from piglet jejunum, which, in turn, enables massive accumulation of electrolytes in the intestine (10). Moreover, it also elicits intestinal mucosal immune responses and enhances inflammatory cytokine production, which damages the small intestine (11). Furthermore, TGEV infection promotes apoptosis and/or necrosis of the small intestinal epithelial cells, which eventually undergoes villous atrophy, disrupts nutrient absorption, produces fatal watery diarrhea and dehydration in piglets, and lead to death (12). Along with the aforementioned pathological changes *in vivo*, TGEV also induces cytopathic effects (CPE) when introduced to cell cultures *in vitro* (13, 14). In particular, TGEV infection accelerates ST cell apoptosis (14–16). p*APN* is the main receptor of TGEV (17), which enters cells *via* the endocytic pathway, involving clathrin and caveolin, assisted by the epidermal growth factor receptor (*EGFR*) and transferrin receptor 1 (*TfR1*) (18, 19). Multiple studies reported the significance of DNA methylation in modulating *pAPN* gene expression (20, 21). In addition, iron metabolism also exhibits a close relation with DNA methylation. Hence, iron levels and status can also affect DNA methylation (22). Unfortunately, there are limited systematic studies on alterations in DNA methylation and gene expression patterns of TGEV infection-related genes. Here, we comprehensively analyzed TGEV infection-induced alterations in methylation and transcriptome of ST cells, using simplified methylation sequencing (RRBS) and RNA-seq techniques. We successfully identified candidate genes and their biological processes that are modulated by TGEV infection and observed their distinct DNA methylation and expression patterns. Our research will add to the growing knowledge of *in vitro* epigenetic and transcriptomic alterations associated with TGEV infection, contribute to the screening of TGEV resistance genes and genetic markers, and enhance the understanding of mechanism on TGEV resistance of piglets.

## Materials and Methods

### Selection of TGEV Processing Time for Sequencing Samples

Porcine testicular cells (ST cells) were purchased from China Center for Type Culture Collection (Wuhan, China) and TGEV were preserved in our laboratory. In this study, we first optimized the viral treatment duration. To do this, ST cells were challenged with TGEV for 0 h, 12 h, 24 h, 48 h and 72 h, respectively. The optimal viral treatment duration was selected *via* detection of CPE generation and real-time PCR detection of viruses. The cells were then inoculated into 6-well plates, and allowed to reach 50% confluency, before introduction of 0.1 MOI viral load to the cells. *TGEV N* gene expression was then assessed *via* fluorescence quantitative PCR to confirm cellular incorporation of the virus.

### Sample Preparation and Nucleic Acid Isolation

Sequencing samples were divided into control and TGEV-infected groups, with 6 biological replicates in each group. To prepare for sequencing, 5 × 10^4^ cells/ml were plated into 6-well plates, cultured overnight, and inoculated with 0.1 MOI virus. The control group was provided with equal volume of phosphate buffer saline (PBS), instead of viral suspension. Cells were harvested after 48 h of treatment and sent out for library construction and sequencing by the Beijing Novo Gene Technology Co., Ltd.

Total RNA and genomic DNA were extracted with Trizol (Thermo Fisher Scientific, USA) and QIAamp DNA extraction kit (Qiagen, Germany), respectively, following kit directions. The RNA and DNA samples were then tested for concentration and purity (A260/A280 within 1.8–2.0 and A260/A230 ≥ 2.0) with the ND-1000 Nanodrop apparatus (Thermo Scientific, USA). Additionally, RNA integrity tests (RIN ≥ 7.0 and 28S/18S ≥ 0.7) were performed with an Agilent 2100 bioanalyzer (Agilent Technologies, USA). The samples that passed quality inspection were sent out for the construction of high-throughput sequencing libraries.

### Methylation Library Generation, Sequencing, and Data Analysis

Upon passing the quality test, lambda DNA (negative control) was introduced and the samples were lysed with methylation-insensitive restriction enzyme MspI (recognition site CCGG), followed by end-repairing, A-tailing, and ligating with sequencing adapters that consisted of methylated cytosines. Next, we selected DNA fragments with 40–220 bp insert lengths *via* gel cutting (23) and treated them with bisulfite using the EZ DNA Methylation Gold Kit (Zymo Research, USA), which allowed unmethylated C to convert into U (which changes to a T upon PCR amplification), while methylated C remained unaltered. Following PCR amplification, the final DNA library was obtained, and quantification was done with Qubit 2.0 (Thermo Scientific, USA), before dilution to 1 ng/μl and subsequent detection of the insert length using Agilent2100 (Agilent Technologies, USA). Lastly, the library effective concentration was assessed by quantitative PCR (library effective concentration > 2 nM).

The libraries that passed quality inspection were subjected to Illumina HiSeq/MiSeq sequencing. Sequencing is typically done *via* synthesis. In short, four fluorescently labeled dNTPs, DNA polymerases, and adaptor primers were added to the sequenced flow cells for amplification, and when each sequencing cluster extended the complementary chain, the corresponding fluorescence was released upon introduction of fluorescently labeled dNTP. The sequencer then transformed the light signal into the sequencing peak *via* a computer software by capturing the fluorescence signal, thereby obtaining the fragment sequencing information.

The sequencing adapters and low-quality fragments of the sequencing data were first truncated and the subsequent analyses were based on clean data. To obtain methylation data, we employed Bismark (bottom call Bowtie2) (24) for alignment analysis of the reference genome Sscrofa11.1 (https://www.ncbi.nlm.nih.gov/genome/?term=pig). The reliability of the methylation site levels was assessed and analyzed in subsequent analyses, based on the results of the Bismark's methylation site detection. We then selected two thresholds (25, 26): (1) sequencing depth ≥ 5; (2) q-value ≤ 0.05 in order to find accurate methylation sites. The methylation degree of a single cytosine was evaluated as the ratio of methylation reads to the read number that detected cytosine. We employed the DSS analysis software for DMR (differentially methylated regions) and DML (differentially methylated loci) analyses (27–29). Adjoining DMRs were merged when the distance between the two was <100 bp.

### RNA-Seq Library Generation and Sequencing

PolyA tails were added to 3 μg of total RNA samples using Oligo (dT) beads, and arbitrarily divalent cations interruptions (prepared in NEB Fragmentation Buffer, NEB, Beijing, China) were introduced. The first strand cDNA synthesis was done with M-MuLV reverse transcriptase system, fragmented mRNA (template), and random primers. This was followed by RNA strand degradation with RNaseH (NEB, Beijing, China), and subsequent second cDNA strand generation was done with dNTPs in a DNA polymerase I (NEB, Beijing, China) system. Next, the purified double-stranded cDNA underwent end-repair, A-tail addition, and ligation with sequencing adapters. AMPure XP beads (Beckman Coulter, Beverly, USA) were employed for the screening of 250–300 bp cDNA, followed by PCR amplification, and an additional purification with AMPure XP beads (Beckman Coulter, USA) was completed to obtain the final RNA library. Library qualification was then done with Qubit2.0 Fluorometer (Thermo Scientific, USA) and Agilent Bioanalyzer 2100 (Agilent Technologies, USA), and sequencing was done with the Illumina HiSeq-PE150 high-throughput sequencing platform.

### RNA-Seq Data Qualification and Gene Expression Quantification

To assess data reliability, we filtered the raw data. To do this, we eliminated reads with adapter, N (indicating that the base data was not available), and low quality (containing bases with Qphred ≤ 20 and making up >50% of the entire read length). Meanwhile, the Q20, Q30, and GC content calculations were performed to clean up the data. As a result, all subsequent analyses used clean data and were deemed as high quality. We next aligned the clean reads to the reference genome Sscrofa11.1 (https://www.ncbi.nlm.nih.gov/genome/?term=pig) using TopHat2 (30). The number of reads per gene was computed with the HTSeq program (31). The fragments per kilobase of transcript sequence per million base pairs for all genes were established *via* the gene lengths and counts of reads mapped to the corresponding genes. Differentially expressed genes (DEGs) analyses of TGEV-inoculated controls were performed using DESeq from the Rpackage (32). The subsequent p-values were corrected with the Benjamini and Hochberg formula to eliminate false discovery rates. Genes with corrected *P*-value < 0.05 and | log2 fold change | > 0.29 were deemed as DEGs.

### Gene Set Enrichment Analysis (GSEA) and Functional Annotation

The biological processes and pathways related to TGEV infection were recognized *via* the GSEA software (33). Pathways possessing FDR q values < 0.2 were regarded as significant. Gene ontology (GO) enrichment analysis was achieved *via* GOseq, according to the Wallenius non-central hypergeometric distribution in the R package (34). GO terms, with an adjusted *p*-value <0.05 were deemed as being obviously enriched. We detected statistical enrichment of KEGG database-based genes with the KOBAS software (35). Networks with normalized *p*-values <0.05 were regarded as significant.

### Verification of the RNA-Seq Data *via* Quantitative Real-Time PCR (qRT-PCR)

ABI 7500 quantitative PCR instrument (Applied Biosystems, FosterCity, CA, USA) and a fluorescence quantification kit (Vazyme) were used to perform qRT-PCR assays. In brief, qRT-PCR was done with 20 μL of sample that contained 10 μL SYBR Green Real-time PCR Master Mix (2 ×), 0.4 μL Reference Dye II (50 ×), 0.4 μL Forward Primer, 0.4 μL Reverse Primer, 2.0 μL cDNA, and 6.8 μL RNase free ddH_2_O. The amplification program was as follows: 95°C for 15 s, 40 cycles of 95°C for 5 s, 60°C for 30 s. *GAPDH* gene was employed as the endogenous control. The utilized primer sequences are presented in Supplementary Table 1. Three replicates were tested by qRT-PCR in each group, and the relative gene expression was computed *via* the 2^−ΔΔCt^ formula (36).

## Results

### TGEV-Infected ST Cell Assay

**Figure 1:**
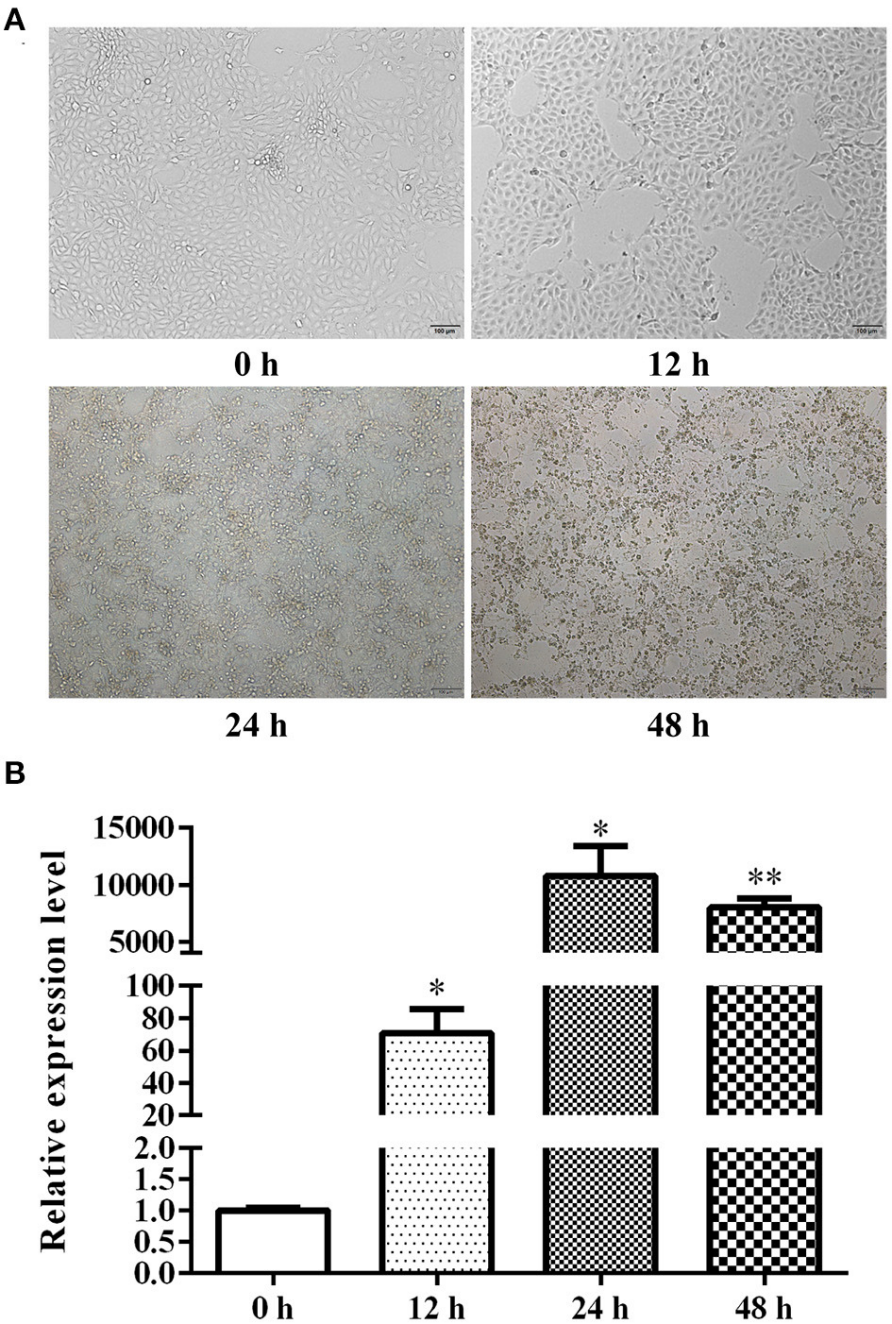
ST cell CPE at different stages of TGEV infection **(A)** and relative TGEV N mRNA levels at different stages of TGEV infection **(B)**. ^*^P < 0.05; ^**^P < 0.01.

TGEV was introduced to cells for 0, 12, 24, 48, and 72 h and CPE was assessed. Based on our results, almost all cells died after 72 h of TGEV infection. In addition, a small number of cells displayed slight cell morphological rounding, cytoplasmic granular degeneration, cell detachment, and other CPE after 24 h of inoculation. With extended inoculation time, CPE gradually became more obvious, and over 95% of cells showed signs of obvious CPE by 48 h of viral inoculation (Figure 1A). We further revealed that with the prolongation of viral treatment time, the virus proliferated, reached its growth peak at 24 h, and then slightly declined at 48 h (Figure 1B). Given these evidences, we selected 48 h to be the optimal 0.1 MOI viral treatment time for subsequent experimentations.

### The DNA Methylome Profile in TGEV-Infected ST Cells

To assess alterations in the DNA methylation patterns after TGEV treatment, four samples and four controls were tested by RRBS after TGEV treatment. We obtained a mean of 41.6 million pure readings per sample, and the mean bisulfite conversion from C to T was >99.1% (Supplementary Table 2). On average, 65.4% of the clean reads were mapped to the pig reference genome (Supplementary Table 2). We separately counted the coverage of C-sites, and these regions covered an average of 48.9% CpG, 1.33% CHG, and 0.96% CHH cytosine in the pig genome (Supplementary Table 3). Since DNA methylation occurred primarily on CpG cytosines (meaning 48.9% methylated CpG, 1.33% methylated CHG, and 0.96% methylated CHH) (Supplementary Table 3). For this study, our focus was on the degree of CpG cytosines methylations. There was bimodal distribution of CpG cytosine methylation levels in the test samples (Supplementary Figure 1), which was in accordance with prior reports on porcine tissues and human cells (23, 37).

**Figure 2:**
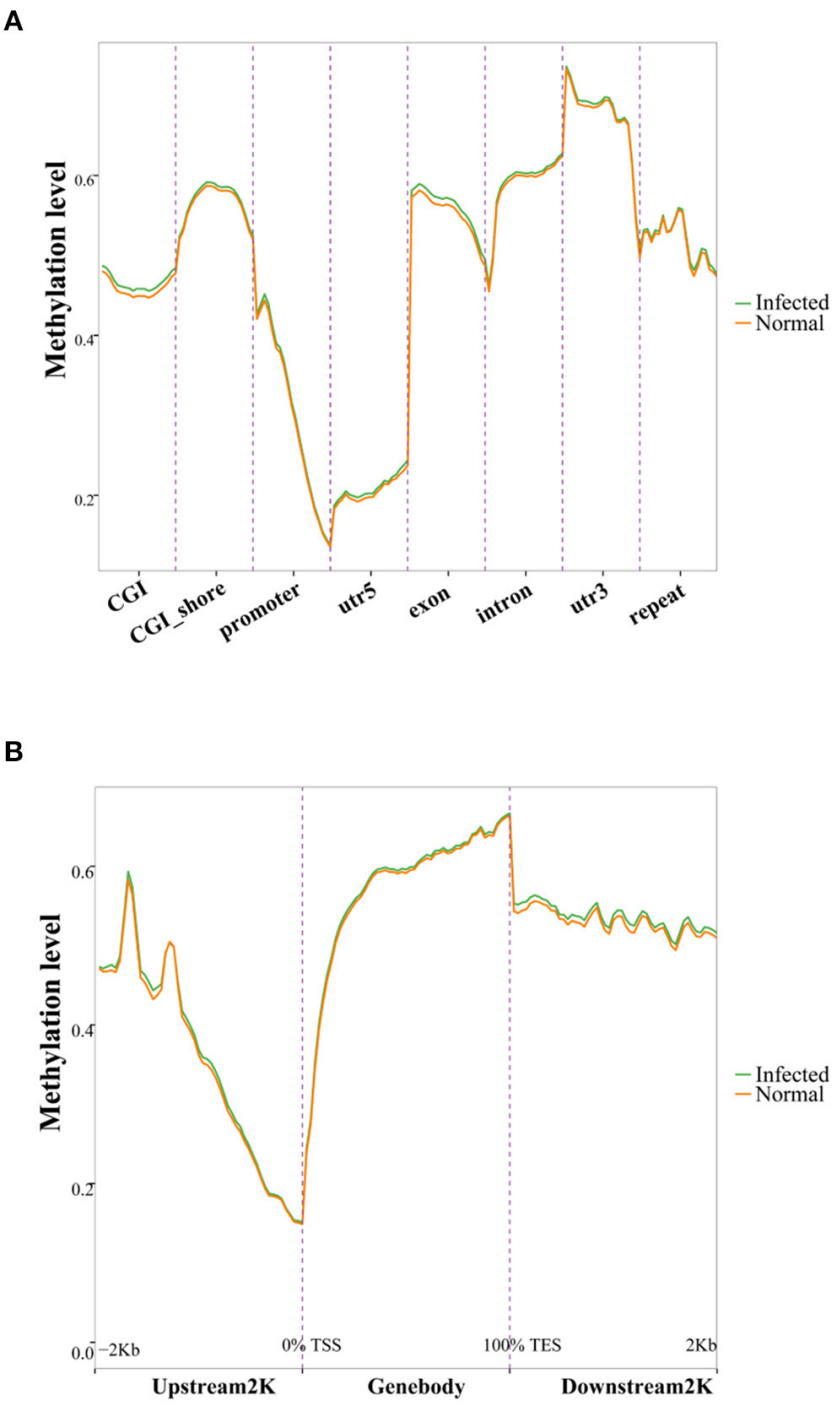
DNA methylation distribution among various genomic elements (GEs) **(A)** and up- and downstream of genes **(B)**. GEs (CGI, CGI shore, promoter, UTR5, exon, intron, UTR3, and repeat) were separated into 20 equal bins, and the mean methylation degree was computed from corresponding GE values. The upstream 2 K, gene bodies, and downstream 2 K regions were separated into 50 bins, and the methylation statuses were averaged. CGI, CpG island; UTR5, 5'-untranslated region; UTR3, 3'-untranslated region.

To elucidate the possible impact of TGEV infection on DNA methylation, we conducted principal component analysis (PCA). Based on our results, TGEV-infected samples formed clusters that were clearly separated from controls (Supplementary Figure 2). To assess alterations in CpG methylation across genomic environments, we calculated average methylation levels across 8 genomic environments. The promoter and UTR5 regions had reduced methylation status compared to other genomic environments (Figure 2A). Moreover, the methylation levels dropped drastically toward the transcription start site, while rising toward the gene body (Figure 2B). The altered DNA methylation pattern that we observed at the transcription start sites was similar to prior reports in other types of cells and tissues (38–40).

**Figure 3:**
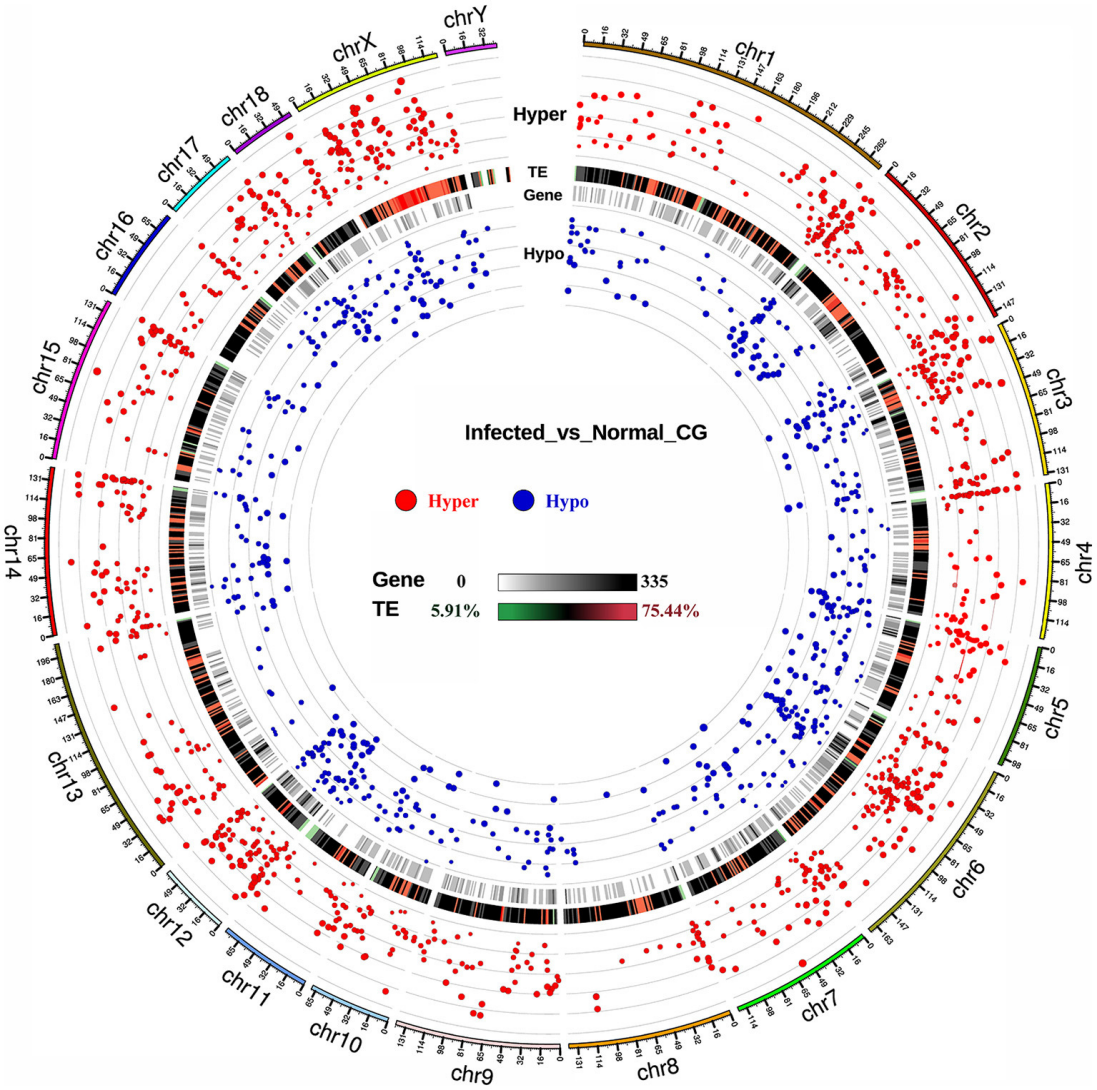
DMR distribution across pig genome. The outermost circle refers to autosomes and the X chromosome. Red and blue dots represent hyper- and hypo-methylated DMRs, respectively. Dot sizes are proportional to the methylation degree between groups. TE, transposable element.

To evaluate TGEV infection-mediated alterations in the DNA methylome, we conducted differential methylation analysis using a smoothing approach. Overall, 1764 DMRs were recognized among the TGEV-treated controls. Among them, 1118 DMRs were highly methylated and 646 had low methylation, as opposed to controls (Supplementary Table 4). Supplementary Figure 3 illustrates the DNA methylation levels in DMRs as well as the differences between both groups. The DMR lengths ranged from 50 to 200 bp (Supplementary Figure 4) and its distribution was mostly in the CGI, CGI shore, promoter, exon, intron and repeat regions (Supplementary Figure 5). Using the areaStat values, we examined the DMR significance and distribution on specific chromosomes in the form of circos plots (Figure 3). The recognized DMRs were found in the promoters of 331 genes and gene bodies containing 1586 genes (Supplementary Table 5). Functional annotation of DMR-related genes demonstrated significant involvement in various molecular functions including “binding (GO: 0005488, 203 genes)” (Supplementary Table 6), “Metabolic pathways (ssc01100),” and “Tight junction (ssc04530)“),” relative to controls.

### DEGs Analysis

4 TGEV-treated ST cell samples and four controls samples were utilized for RNA-seq analysis (Supplementary Table 8). We obtained ~367.9 million raw reads, using next-generation RNA sequencing. This included 360.9 million clean reads that underwent quality control analysis, with a mean of 45.12 million clean reads per sample (Supplementary Table 8). Alignment analysis revealed that ~347.2 million reads (96.2%) were identified in the pig genome, among which 334.8 million reads (92.78%) were distinctly mapped (Supplementary Table 9). Reads distribution across the pig genome showed that 91.94% of reads came from exons and 8.06% from introns and intergenic regions (Supplementary Table 10), confirming the effectiveness of our data in reflecting the genomic gene expression profiles of our analyzed samples. PCA results also indicated that the TGEV-infected samples formed clusters that were clearly separated from control samples (Supplementary Figure 6).

**Figure 4:**
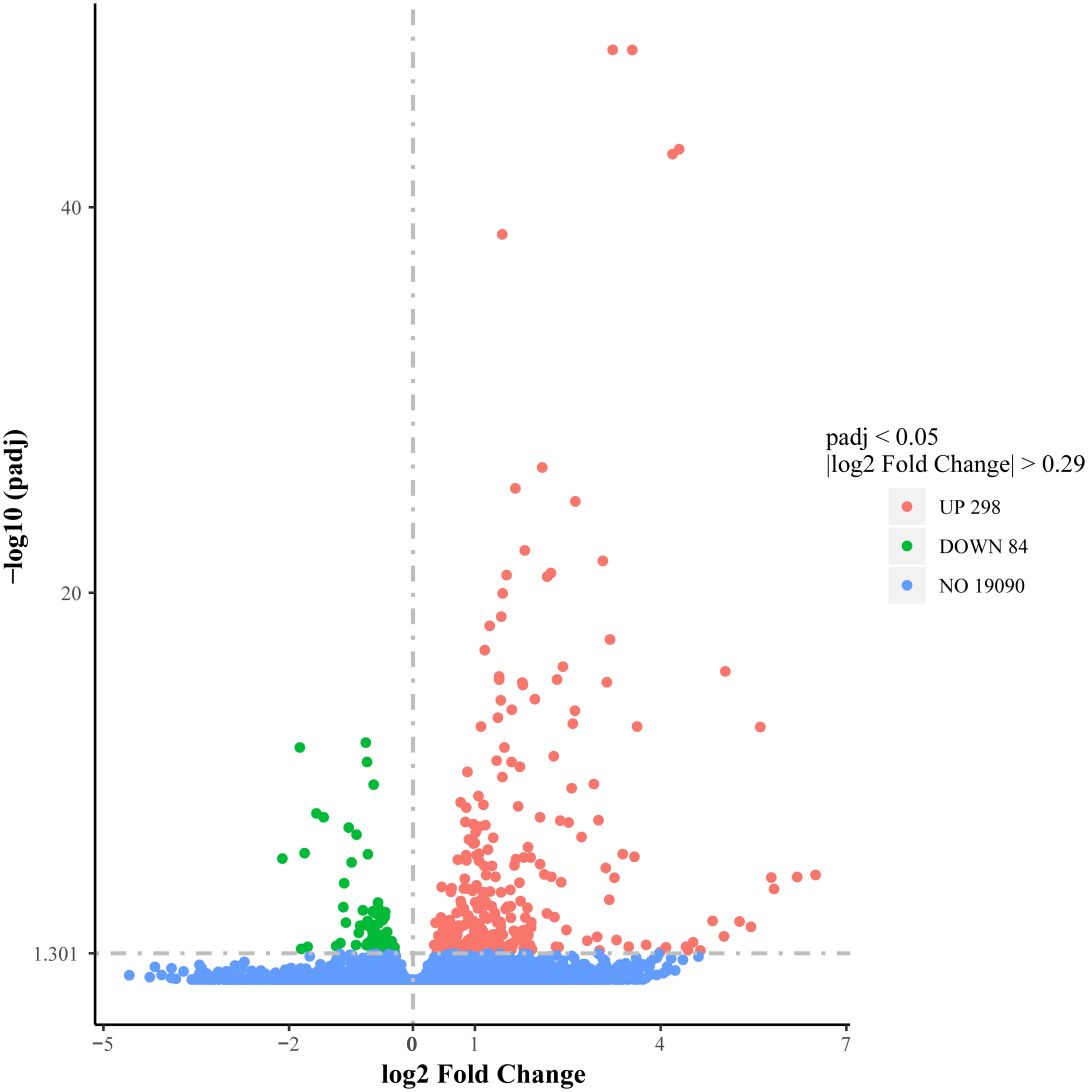
Volcano plot illustrating differential gene expression between TGEV-inoculated and control groups. Red dots denote markedly elevated genes; green dots, markedly reduced genes; black dots, no marked alterations.

**Figure 5:**
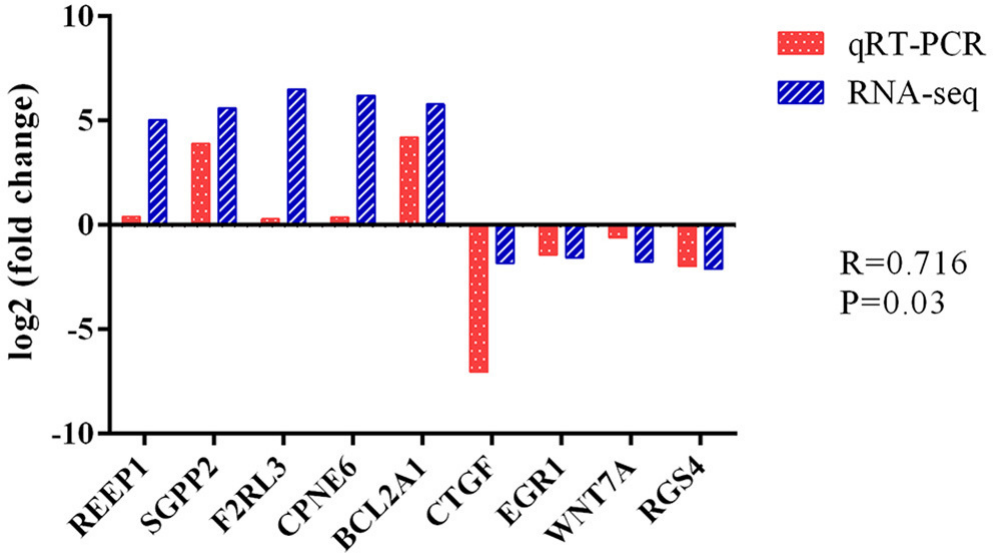
Differential expression of 9 selected genes, as evidenced by RNA-seq and qRT-PCR. Fold changes are presented as ratio of gene expression in TGEV-inoculated vs. control samples. Red and blue bars denote qRT-PCR and RNA-seq results, respectively.

To evaluate the DEG differences between TGEV-inoculated and control samples, DEG analysis was performed. Overall, 382 DEGs were identified (| log2 Fold Change | > 0.29, corrected *P* < 0.05), including 298 elevated and 84 reduced gene expressions (Figure 4) (Supplementary Table 11). We next validated the DEG gene identification of nine select genes (*REEP1, SGPP2, F2RL3, CPNE6, BCL2A1, CTGF, EGR1, WNT7A*, and *RGS4*) using qRT-PCR. Based on our data, both RNA-seq and qRT-PCR provided similar results (Figure 5), carrying a Pearson correlation coefficient (PCC) of 0.716 (*P* = 0.03), suggesting that the RNA-seq data was highly reliable and accurate.

GSEA was employed for the detection of TGEV infection-related biological processes and signaling pathways. Based on our results, the signal transduction in absence of ligand, defense response to virus, viral response, and endocytotic pathways were significantly upregulated (Supplementary Figures 7A,B, Supplementary Table 12), whereas the ribonucleoprotein complex generation, rRNA metabolic network, and ribosome production were markedly down-regulated in TGEV-infected samples (Supplementary Figures 7A,C, Supplementary Table 12). We thus demonstrated that TGEV infection suppresses protein and nucleic acid generation *via* interaction with ribosomes. Additionally, a sub-category of DEGs were shown to be strongly enriched in the defense response to virus and ribosomal generation categories (Supplementary Figures 8A,B), thereby indicating their role in inducing cellular apoptosis, in response to TGEV infection.

### Integrative Analysis of Transcriptome and Methylation Information

**Figure 6:**
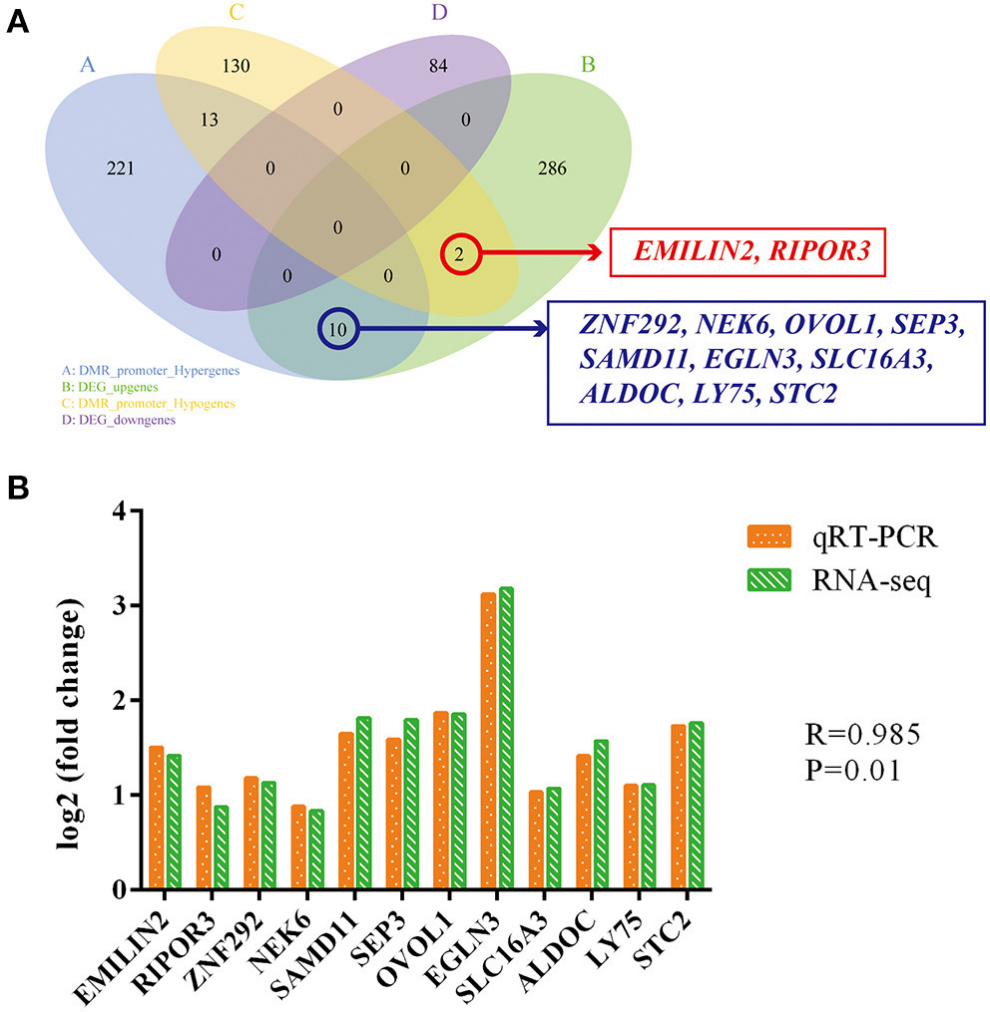
Venn diagram of DMR-related genes and differential genes, as evidenced by transcriptome analysis **(A)**. Expression alterations of 12 sieved genes, as evidenced by RNA-seq and qRT-PCR. Fold changes are presented as ratio of gene expression in TGEV-inoculated versus control samples. Yellow and green bars denote qRT-PCR and RNA-seq results, respectively **(B)**.

To examine the DNA methylation-mediated modulation of gene expression, we performed a comprehensive analysis of DMRs and gene expression profiles. Generally, DNA methylation at a promoter site renders a gene dormant, i.e., it cannot be transcribed (41). Therefore, we assessed the association between promoter methylation status and DEG profile. We identified 2 promoter DMRs that downregulated expression of DEGs *EMILIN2* and *RIPOR3* (Figure 6A, Supplementary Table 13). In the meantime, we also identified 10 DMRs that upregulated certain DEG expressions, namely, *ZNF292, NEK6, OVOL1, SEP3, SAMD11, EGLN3, SLC16A3, ALDOC, LY75*, and *STC2* (Figure 6A, Supplementary Table 13). We next performed qRT-PCR quantitative validation of these 12 genes and confirmed that the RNA-seq data and qRT-PCR analysis produced consistent DEG profiles (Figure 6B), carrying a PCC of 0.985 (*P* = 0.01), again suggesting that our sequencing data was highly reliable and accurate. In a majority of the cases, the changes in gene expression coincided with alterations in their methylation status at gene bodies (42). We recognized 31 DEGs that contained different degrees of methylation in their gene bodies (Supplementary Table 14). Our data indicated a strong positive correlation between DNA methylation and gene expression in the most of these DEGs at gene bodies (20 out of 31) (Supplementary Figure 9).

## Discussion

In addition to the *in vivo* assays and intestinal organoid culture systems (43), most of the studies against TGEV were performed *via* TGEV infection of cell lines *in vitro*. TGEV can infect a variety of cell lines, and among the more susceptible cells are the PK-15 and ST cells (44). TGEV propagation within PK-15 and ST cells was reported to induce CPE in both cell lines, with no particular difference in the duration of CPE and viral titer. Studies revealed that TGEV accelerates apoptosis of ST, but not intestinal epithelial cells (15).

The molecular events surrounding viral infection of host cells involves a dynamic process of change. In fact, Brunborg et al. (45) observed that inflammatory factors like *interleukin (IL)-6* and *-8* transiently increased after 3 h post infection and rose again after 24 h of swine fever virus SFV infection, the levels remained persistently elevated for a prolonged time. Imam et al. (46) reported that the HIV-based Nef protein suppresses lncRNA NRON expression during early infection stage, while elevated Vpu protein levels in later stages of infection raises NRON expression. Based on these evidences, viral infection duration exerts a direct effect on its interaction with host cells. Here, we inoculated ST cells with TGEV for 0, 12, 24, 48 and 72 h to observe virus-induced lesions and detect the virus. Based on our results, virus titers increased at 12 h after TGEV inoculation. However, there was still no significant difference in cytopathic effect between the TGEV-inoculated and MOCK groups, which is in accordance with reports of the effects of early viral infection. However, the virus titers were significantly increased at 24 h after infection, and the degree of CPE was mild. After 48 h of TGEV treatment, the virus titers were even more elevated, and almost all cells exhibited CPE, which was comparable to earlier reports on the features at later stage after viral infection. At this point (72 h), almost all cells died, and RNA could not be extracted for qRT-PCR detection. Hence, in our subsequent experiments, we chose to treat ST cells with TGEV for 48 h to represent the stage of complete viral infection, based on two indicators: CPE and viral load.

Epigenetic modification refers to alterations in the chromatin structure, without DNA sequence changes (47). Epigenetic changes often occurs due to environmental and behavioral alterations such as diet and temperature (48). Multiple environmental factors manipulate the degree of epigenetic modifications, and thus promote transmission of epigenetic information (like cellular apoptosis) to offspring (49, 50). Xiao et al. (51) reported that, methylation of the mC-5 site in the *MUC2* promoter inhibited the binding of Yin Yang 1 (YY1) to the promoter, down regulated the expression of *MUC2* and increased the susceptibility of piglets to porcine epidemic diarrhea virus (PEDV). We also have confirmed abnormal methylation at the *AQP3* promoter reduce its expression in PEDV-infected piglet jejunum (52). Zhang et al. (53) suggested that porcine reproductive and respiratory syndrome virus (PRRSV) vaccination in sows induces DNA methylation changes in genes and DNA methylation changes occur through intergenerational transmission. Weber et al. (54) concluded that DNA methylation can control transcription of porcine endogenous retroviruses (PERV) on the *sus scrofa* genome, and PERV 5'LTR hypo-methylation can serve as a marker of active provirus. Given that methylated DNA directly modulates normal and pathophysiological environment, gaining extensive knowledge of the DNA methylation patterns across genomes is critical to discovering the true significance of epigenetics. Hence, we examined genome-wide DMRs after TGEV infection in ST cells. In all, we identified 1764 DMRs and analyzed their localization on *sus scrofa* genome. Our revealed that a majority of DMRs (1118 of 1764) were hypermethylated, suggesting that TGEV induces widespread DNA methylation in ST cells. We also revealed that DMRs-related genes were highly enriched in metabolic pathways and tight junction processes, thus, TGEV likely exerts its infectious role through these two physiological processes. It was demonstrated that TGEV infection can induce epithelial-mesenchymal transition (EMT) in IPEC-J2 cells, increase ETECK88 adhesion to the intestine, and promote double infection, thereby aggravating diarrhea in piglets (55). Our dataset provides an additional layer of epigenetic insight into TGEV pathogenesis as well as regulatory mechanisms of host immune response.

As early as 1988, Sirinarumitr et al. (16) determined that TGEV can promote ST cell apoptosis using agarose gel electrophoresis, electron microscopy, and deoxyribonucleopropionate terminal transferase-mediated nick end labeling. Subsequently, Eleouet et al. (14, 56) infected human rectal tumor cells with TGEV and demonstrated that caspase-3,−6,−7,−8 and−9 were stimulated after infection, suggesting caspase involvement in TGEV-mediated apoptosis. Ding et al. (57). reported that, in TGEV-infected PK15 cells, the FasL-mediated apoptotic pathway was activated. Additionally, TGEV markedly reduced levels of the anti-apoptotic factor *Bcl-2* and promoted the transfer of Bax to mitochondria, where it stimulated the mitochondrial apoptotic network, thereby initiating a cascade of activities that included cytochrome c release and caspase (9 and 3) induction, followed by apoptosis activation. In our study, we demonstrated that *BCL2A1* (BCL2 Related ProteinA1, a pro-apoptotic modulator, *BCL2* family) was significantly upregulated after TGEV infection (Supplementary Table 11), indicating activation of the apoptotic process. Additionally, based on the Huang et al. (58) study, TGEV incorporation can obviously lower both p300/CBP and *MDM2* levels and simultaneously upregulate p53 levels by phosphorylating serine residues at positions 15, 20, and 46 of p53, in addition to transiently activating the p38-MAPK pathway to mediate apoptosis during early infection. Ding et al. (59) observed that TGEV infection promotes accumulation of reactive oxygen species (ROS), which lowers cellular mitochondrial membrane potential, thus activating the p38-MAPK network and p53, which, in turn, induces apoptosis. This study evidences the crucial role of ROS in TGEV-induced apoptosis. In our study, we demonstrated high expression of *IL-16* after TGEV-inoculation of ST cells (Supplementary Table 11), verifying the significance of *IL-16* cytokines in cellular immunity against TGEV infection.

We also performed an extensive evaluation of DMRs and gene expression profiles to delineate the effect of promoter methylation status on DEGs. Most studies suggested that high DNA methylation is inhibitory toward gene expression, and demethylation allows gene re-expression (60). Based on our analysis, we located 2 DMRs within the promoter region, which were negatively correlated with the DEGs Elastin microfibrillar interface localization protein 2 (*EMILIN2*) and RIPOR family member 3 (*RIPOR3*) (Supplementary Table 13). *EMILIN2* was previously recognized as a candidate gene for thrombosis in quantitative trait locus studies in mice and humans (61). It is known to promote angiogenesis by direct association with epidermal growth factor receptor (*EGFR*), which increases *IL-8* production. As a result, human tumors with high *EMILIN2* expression tend to be more sensitive to chemotherapy (62). We demonstrated that *EMILIN2* expression was significantly up-regulated, with significant down-regulation of promoter methylation after TGEV infection in ST cells. *EGFR* is a co-factor of TGEV that can cooperate with *APN* to stimulate both P13K/AKY and MEK/ERK1/2 pathways (18). *EMILIN2* upregulation facilitates EGFR-related extracellular receptor domain 1 interaction with TGEV S proteins, which accelerates TGEV invasion. A comprehensive understanding of TGEV-induced regulation of *EMILIN2* promoter methylation status can facilitate its potential usage as a biomarker for TGEV-induced cell apoptosis.

## Data Availability Statement

Sequencing data has been submitted to NCBI's SRA repository, https://www.ncbi.nlm.nih.gov/sra, under BioProject accession number PRJNA766131.

## Author Contributions

WB and SW conceived and supervised the study. JW and WB designed the experiments. JW and XS performed the experiments. JW and LW analyzed the data. JW, ZW, and WB contributed to the writing of the manuscript. All authors contributed to the article and approved the submitted version.

## Funding

This research was funded by National Natural Science Foundation of China (31972535), Postgraduate Research & Practice Innovation Program of Jiangsu Province (KYCX20_2987), Key Research and Development Project (Modern Agriculture) of Jiangsu Province (BE2019344 and BE2019341), Jiangsu Agricultural Science and Technology Innovation Fund (CX(20)3011), and the Priority Academic Program Development of Jiangsu Higher Education Institutions.

## Conflict of Interest

The authors declare that the research was conducted in the absence of any commercial or financial relationships that could be construed as a potential conflict of interest.

## Publisher's Note

All claims expressed in this article are solely those of the authors and do not necessarily represent those of their affiliated organizations, or those of the publisher, the editors and the reviewers. Any product that may be evaluated in this article, or claim that may be made by its manufacturer, is not guaranteed or endorsed by the publisher.
