# Supplementary material for: Genome-Wide DNA Methylome and Transcriptome Analysis of Porcine Testicular Cells Infected With Transmissible Gastroenteritis Virus
Source: Front Vet Sci. 2022 Jan 13;8:779323. doi: 10.3389/fvets.2021.779323 (PMC8794705; doi:10.3389/fvets.2021.779323)
Supplement: Supplementary file 1 [file Table_1.DOCX]

Supplementary Figures

**
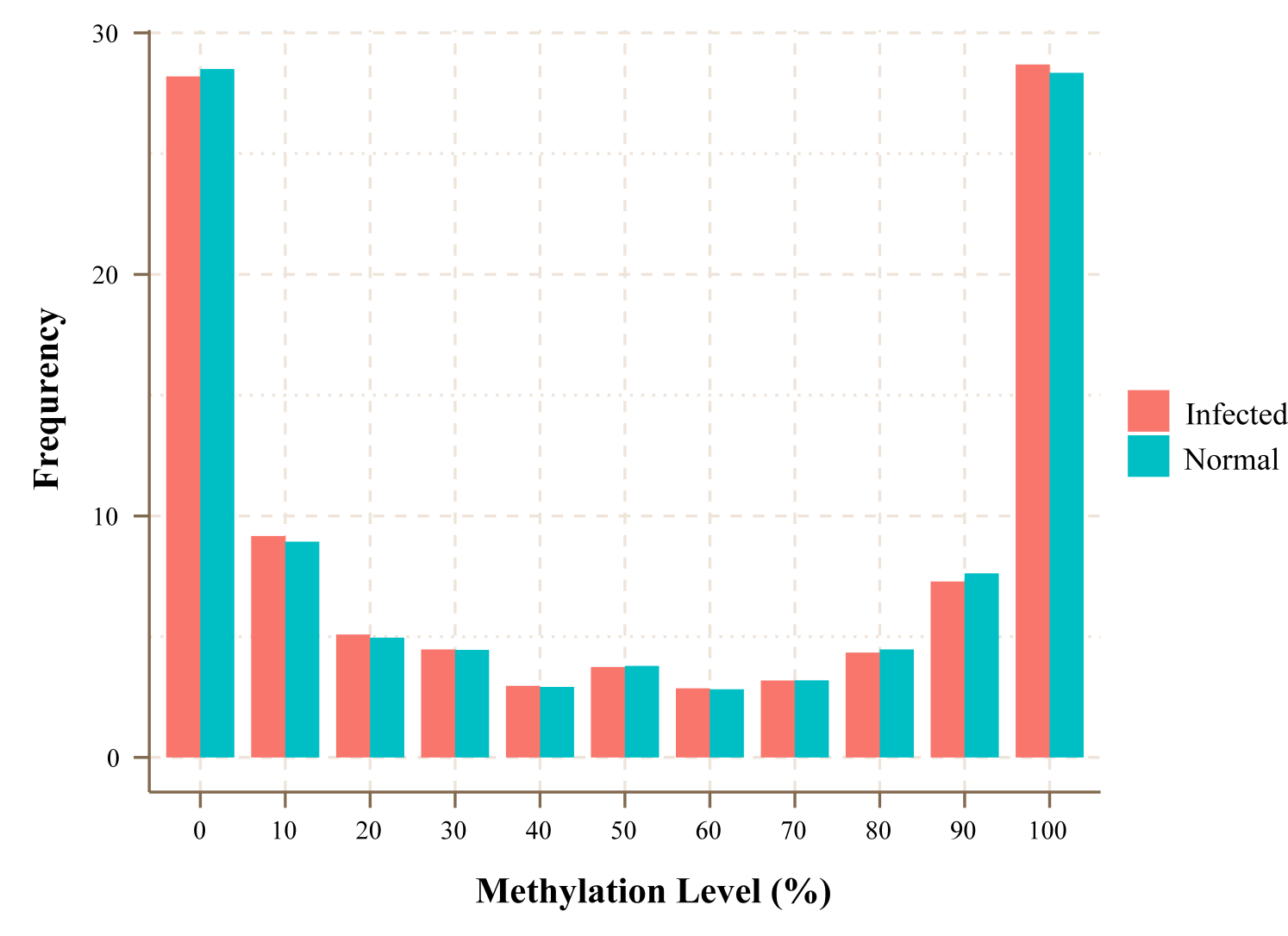
**

**Figure S1** Distribution of CpG cytosine methylation percentage per base of TGEV-infected and control group.


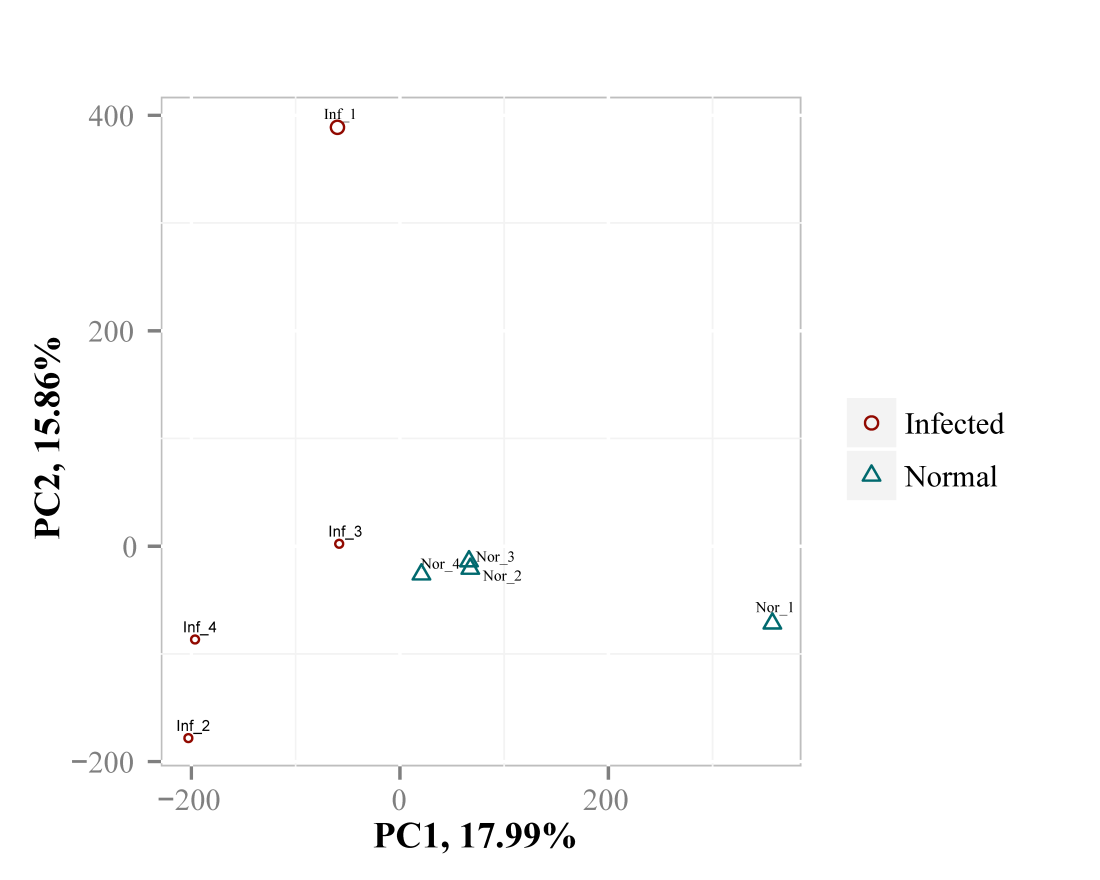


**Figure S2** Principal component analysis based on DNA methylation level of the samples. The cyan dots and red triangles represent TGEV-infected and control samples, respectively.


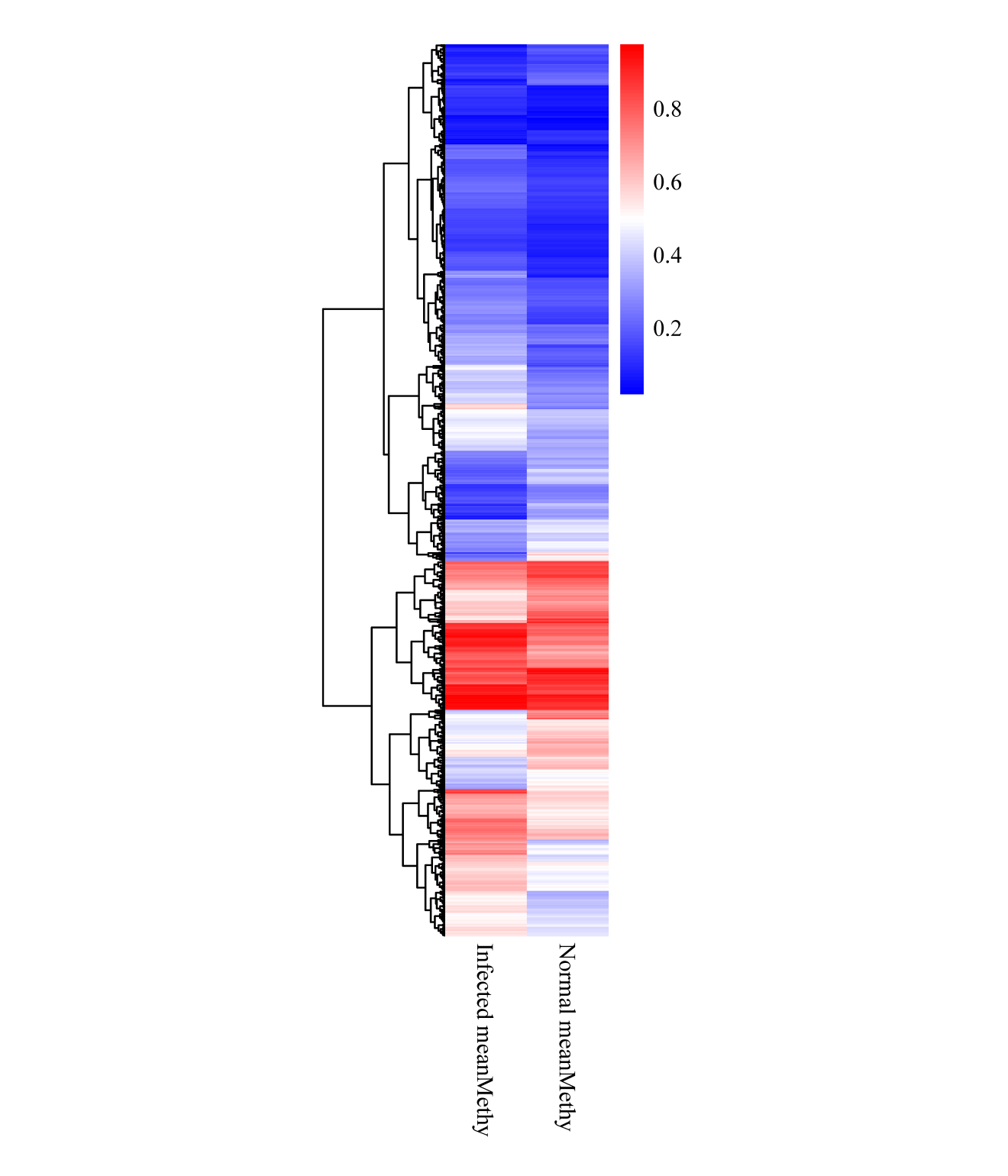


**Figure S3** [Heatmap](https://www.sciencedirect.com/topics/medicine-and-dentistry/heat-map) of [differential methylation](https://www.sciencedirect.com/topics/medicine-and-dentistry/differential-gene-expression) between TGEV-infected and control groups.


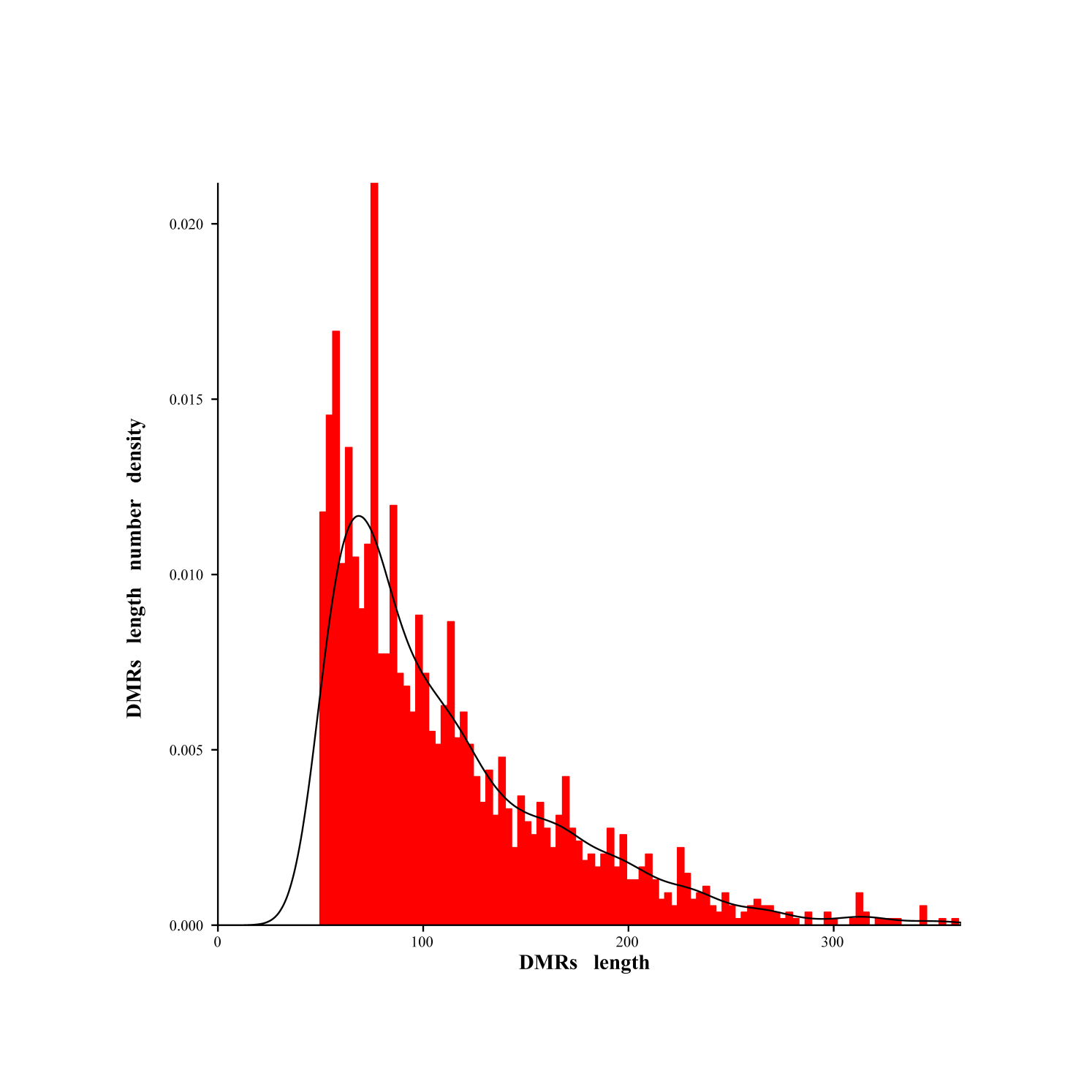


**Figure S4** Distribution of DMRs length. The black line indicates the fitting curve of distribution.


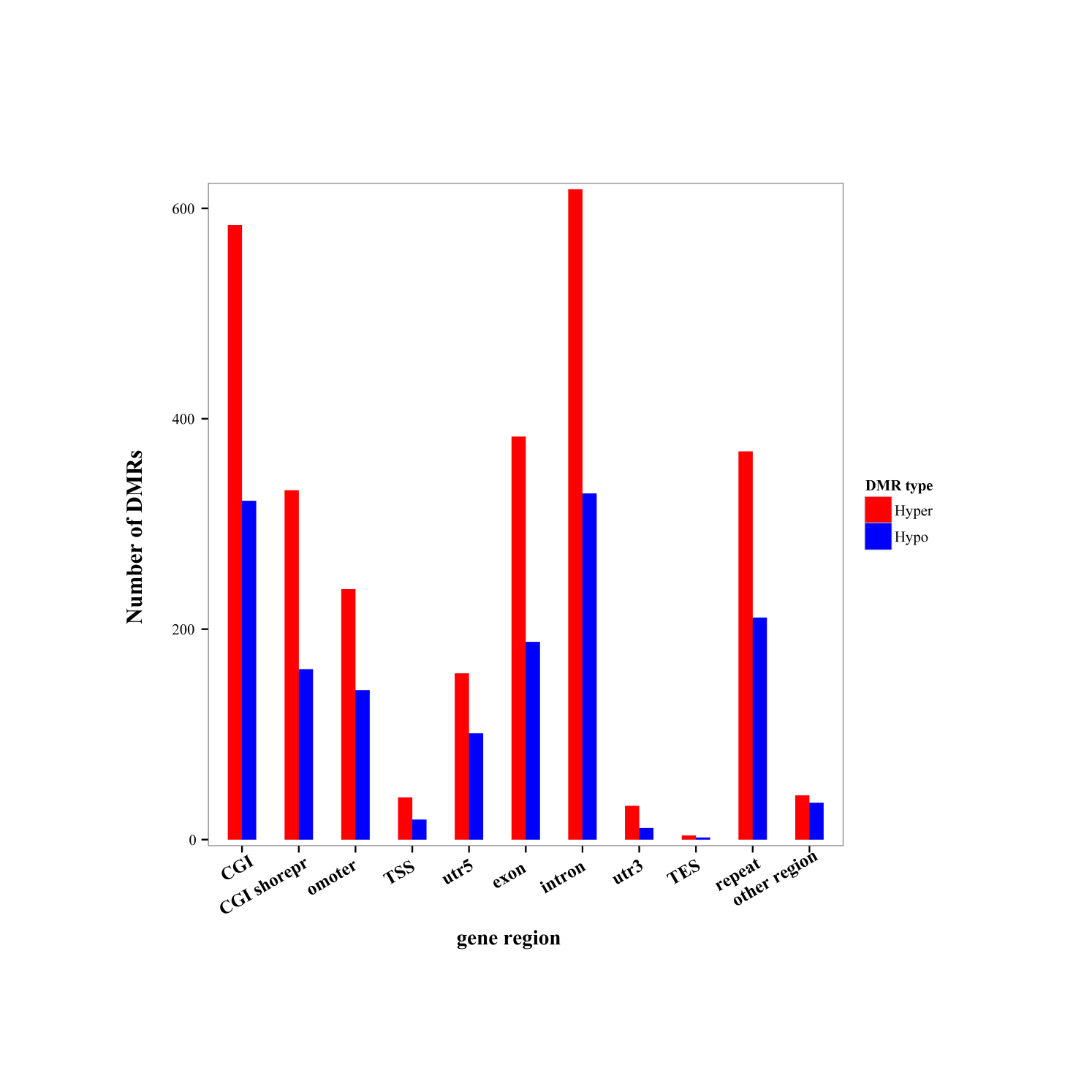


**Figure S5** Number of DMRs mapped to different genomic contexts. The red and blue bars represent hypermethylated and hypomethylated DMRs, respectively. CGI: CpG island; UTR5: 5’-untranslated region; UTR3: 3’-untranslated region.


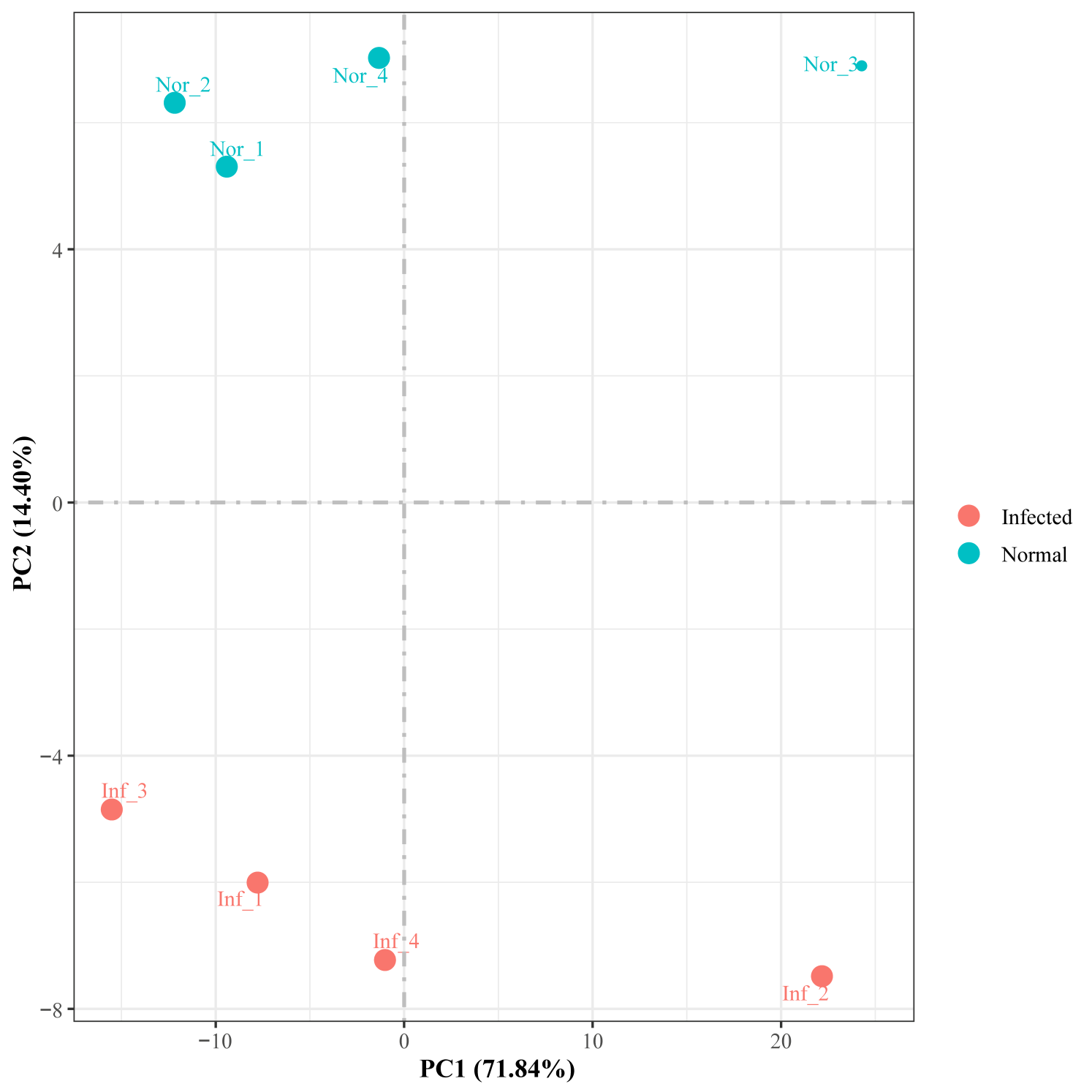


**Figure S6** Principal component analysis of the samples. The cyan dots and red triangles represent TGEV-infected and control samples, respectively.


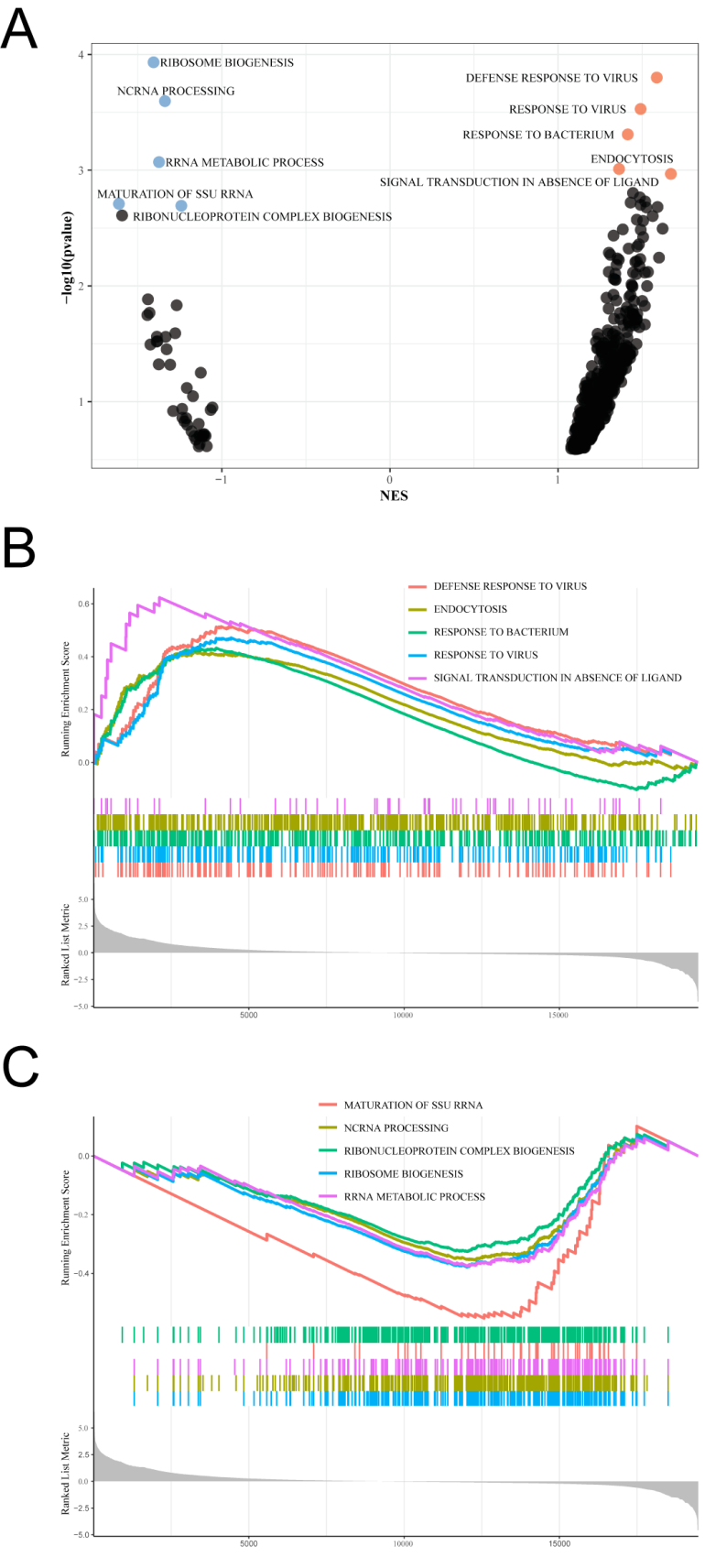


**Figure S7** Gene set enrichment analysis (GSEA) of TGEV-infected cell transcripts (A). GSEA volcano plot, using the MSigDB Hallmark database. FDR versus corrected enrichment score (NES) for each gene set is presented. Blue denotes negative association (B); and red, positive association (C).

**
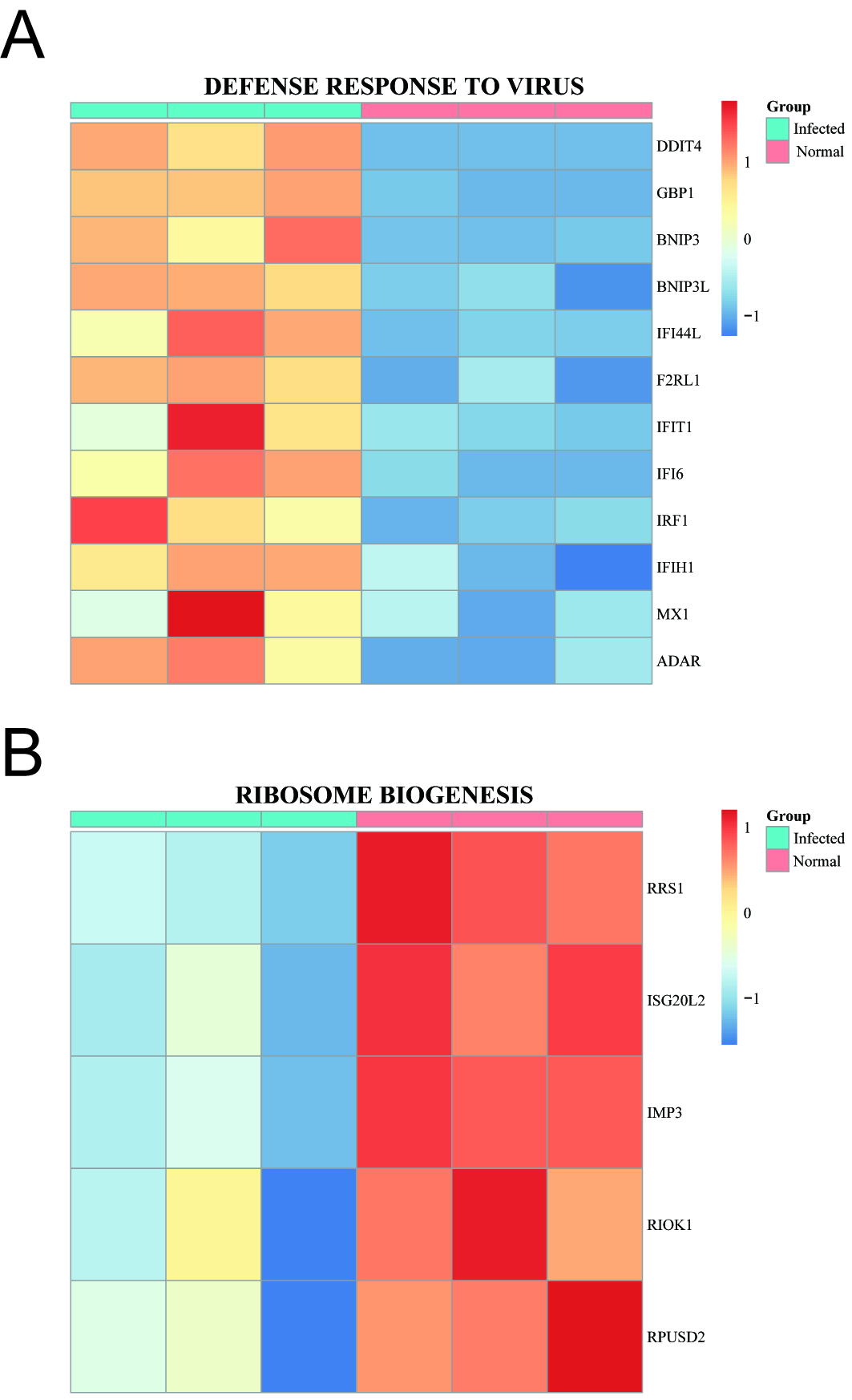
**

**Figure S8** RNA-seq quantified gene expression. Red, high relative gene levels; blue, low relative gene levels. Infected: TGEV-inoculated samples; Control: untreated samples.


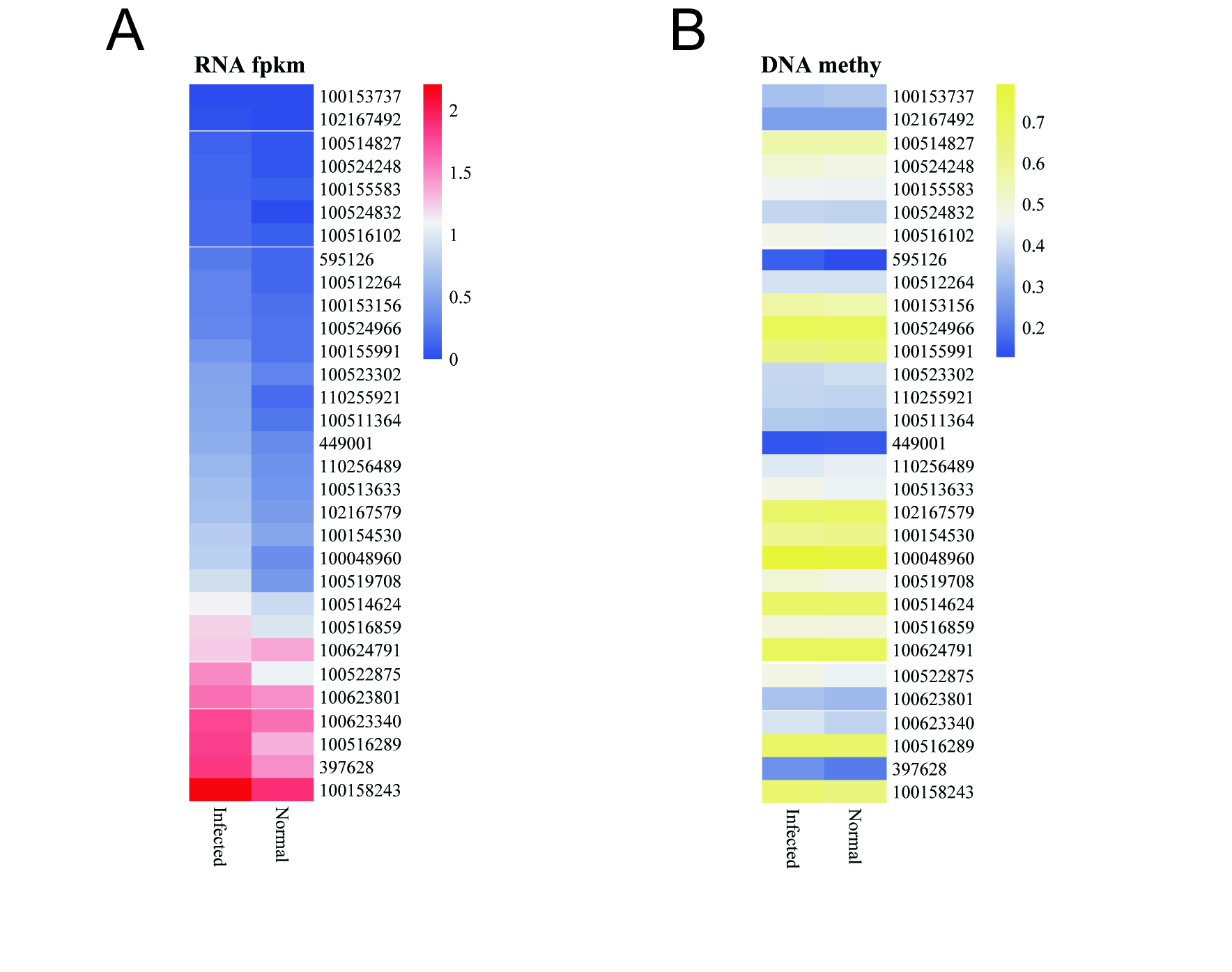


**Figure S9** Hierarchical cluster analysis of methylation level and expression level of overlapping genes in genebody region.
